# Low-Intensity Steady Background Noise Enhances Pitch Fusion Across the Ears in Normal-Hearing Listeners

**DOI:** 10.3389/fpsyg.2021.626762

**Published:** 2021-02-01

**Authors:** Yonghee Oh, Sabrina N. Lee

**Affiliations:** Department of Speech, Language, and Hearing Sciences, University of Florida, Gainesville, FL, United States

**Keywords:** pitch, steady pink noise, stochastic resonance, binaural auditory processing, binaural fusion

## Abstract

Binaural pitch fusion is the perceptual integration of stimuli that evoke different pitches between the ears into a single auditory image. This study was designed to investigate how steady background noise can influence binaural pitch fusion. The binaural fusion ranges, the frequency ranges over which binaural pitch fusion occurred, were measured with three signal-to-noise ratios (+15, +5, and −5dB SNR) of the pink noise and compared with those measured in quiet. The preliminary results show that addition of an appropriate amount of noise can reduce binaural fusion ranges, an effect called stochastic resonance. This finding increases the understanding of how specific noise levels can sharpen binaural pitch fusion in normal hearing individuals. Furthermore, it elicits more pathways for research to explore how this benefit can practically be used to help improve binaural auditory perception.

## Introduction

Stochastic resonance is a phenomenon where a signal transmission in a nonlinear system is enhanced by the addition of an external noise. This effect has been observed in many human sensory functions including visual perception (Kitajo et al., 2003; Sasaki et al., 2006; Aihara et al., 2008), somatosensation (Collins et al., 1996), and auditory perception (Zeng et al., 2000; Chatterjee and Robert, 2001; Allingham et al., 2003; Paglialonga et al., 2010; Tanaka et al., 2010; Martignoli et al., 2013; Oh et al., 2015; Othman et al., 2019). All of these studies have revealed that the addition of a small but optimal level of external noise to each sensory system enhances its response (detection or discrimination) to an input signal, whereas adding large amount causes it to deteriorate (masking).

In the area of auditory perception, findings in healthy auditory system showed that the addition of noise improved auditory phase response (Tanaka et al., 2010), auditory detection performance for complex signals (Oh et al., 2015), and auditory working memory (Othman et al., 2019). Other studies tested damaged auditory systems and also showed that an optimal amount of the noise enhanced auditory modulation sensitivity (Chatterjee and Robert, 2001) and frequency discrimination ability (Zeng et al., 2000) in cochlear implant (CI) users, specifically. Here, the CI is a surgically implanted electronic device (e.g., auditory prosthetic) that is inserted into the damaged cochlea to aid signal transmission through the auditory pathways.

In particular, multiple studies focused their research on a specific form of stochasticity called “suprathreshold stochastic resonance (SSR)” which differs from conventional stochastic resonance in that it does not rely on a weak signal and occurs at a non-zero sensational level of noise intensity. Martignoli et al. (2013) explored the SSR effects of white noise on different levels of spontaneous nerve firings as it related to pitch perception in a model of a healthy cochlea. Other model-based studies also demonstrated that SSR does appear to offer a means of improving fiber spontaneous activity and its signal transmission when CI electric stimuli are combined with noise (Allingham et al., 2003; Paglialonga et al., 2010). All of these studies suggested that one possible common mechanism to explain sensitivity improvement is that an optimal amount of external noise in either near threshold or suprathreshold levels can re-generate spontaneous activity in various classes of auditory nerves (high, medium, and low-spontaneous auditory nerve neurons).

In this study, we investigated this SSR effect on pitch perception in binaural listening conditions, with particular interest in pitch discrimination ability in dichotically presented tones across the ears, which is referred to as binaural pitch fusion in this study. Binaural pitch fusion is the perceptual integration of stimuli that evoke different pitches between the ears into a single auditory image. For a given individual there will be a range of frequencies that they will perceive as one pitch. They may have a broad or narrow fusion range where they either hear many frequencies as one pitch or only a few frequencies as one pitch, respectively. Normal hearing (NH) listeners tend to have fusion ranges spanning 0.1–0.2 octaves across the ears (Thurlow and Bernstein, 1957; van den Brink et al., 1976; Reiss et al., 2017) while hearing impaired (HI) individuals have ranges as wide as 3–4 octaves in pitch (Reiss et al., 2014, 2018; Oh and Reiss, 2018).

One recent study by Oh et al. (2019) suggested that broad fusion is associated with greater difficulty in using voice pitch difference cues to separate a target voice from other interfering voices in both NH and HI listeners. In other words, the broader the pitch fusion, the smaller the benefit from voice pitch differences because broad fusion could lead to abnormal spectral fusion and blending of words from voices of different pitches. In this study, our main question was can we find stochastic resonance effects on binaural pitch fusion, or more specifically, can we use the addition of noise to sharpen binaural pitch fusion? We hypothesized that adding a nonzero noise would reduce the breadth of fusion by enhancing binaural pitch discrimination ability, due to a suprathreshold stochastic resonance effect, in which an optimal amount of added noise results in enhanced signal transmission through binaural auditory pathways. If this method of testing is able to elicit changes to binaural pitch fusion in NH individuals, an interesting path to possibly elicit changes in binaural fusion for HI individuals, who often show broader fusion ranges than in NH listeners, is revealed.

## Materials and Methods

### Subjects

Ten NH adults (nine females) ranging in age from 20 to 39 (mean and std. = 25 ± 6) participated in this study. NH was defined as air conduction thresholds ≤25dB hearing level (HL) from 125 to 8,000Hz. Averaged audiometric thresholds were 7.1 ± 6.5dB HL and 6.7 ± 7.3dB HL for left and right ears, respectively. All subjects were screened for normal cognitive function using the Mini Mental Status Examination (MMSE) with a minimum score of 27 out of 30 required to qualify (Folstein et al., 1975; Souza et al., 2007). Both ethical and methodological approvals were obtained from the Institutional Review Board of University of Florida. All subjects provided written informed consent.

### Stimuli and Procedures

All experiments were conducted in a double-walled, sound attenuated booth (ETS-Lindgren/Acoustic Systems, Texas, United States). Signals were generated at a sampling rate of 44.1kHz with MATLAB (version R2018b, MathWorks, Massachusetts, United States), processed through an RME Babyface Pro sound card (RME Audio, Haimhausen, Germany), and presented over Sennheiser HD-280 Pro headphones (Sennheiser, Sedemark, Germany). Each headphone’s frequency response was equalized using calibration measurements obtained with a Brüel & Kjær sound level meter (Brüel & Kjær Sound & Vibration Measurement A/S, Nærum, Denmark) with a 1-inch microphone in an artificial ear.

All stimuli consisted of pure tones with 10-ms raised-cosine onset/offset ramps. Prior to all experiments, loudness balancing was conducted using a method of adjustment. First, 300-msec tones with octave spacing between 0.125 and 8kHz in the left ear were initialized to “medium loud and comfortable” levels corresponding to a 6 or “most comfortable” on a visual loudness scale from 0 (no sound) to 10 (too loud). Loudness for the right ear was then adjusted for each frequency to be equally loud to a tone in the left ear during simultaneously presentation between the ears, based on subject feedback. Here, all loudness balancing adjustments were repeated with a fine attenuation resolution (0.1–0.5dB steps) until equal loudness was achieved with all comparison sequences within and across ears. The frequencies and order of presentation were randomized to minimize the effect of biases such as time-order error and overestimation of the loudness for high-frequency tones (Florentine et al., 2011). Interpolation (on a dB scale with a linear frequency) was then used to determine appropriate levels for all tone frequencies used in testing. Averaged loudness balanced sound levels were 62.8 ± 4.9 and 63.4 ± 4.7dB SPL for left and right ears, respectively. This loudness balancing procedure was performed to minimize use of level-difference cues and maximize focus on pitch differences as the decision criteria.

For the dichotic fusion range measurement, the method of adjustment (adaptive method) was used. The designated reference ear was presented with the same tone stimulus on every trial, and contralateral comparison ear was presented with a stimulus that varied across trials. Both reference and comparison stimuli consisted of coherent amplitude-modulated (AM) tones (4-HzAM rate with 100% AM depth) with different carrier frequencies that were dichotically presented in a 1500-ms single interval in a two-alternative forced choice paradigm. Subjects were asked to indicate whether they heard a single fused sound (i.e., the dichotic stimuli were perceived to be integrated) or two different sounds in each ear (i.e., the dichotic stimuli were perceptually different). The reference tone frequency for the reference ear was fixed at 2kHz or 3kHz, and comparison tone frequencies were adaptively varied at each trial using a two-down-one-up procedure (Levitt, 1971) to estimate the range of frequency yielding 70.7% correct perceptual fusion between two ears. Four fusion range measurement tasks were interleaved with different initial frequencies in the contralateral ear: 0.5–1.5 octaves above and below the reference frequency, and with different reference ears: left and right ears.

**Figure 1:**
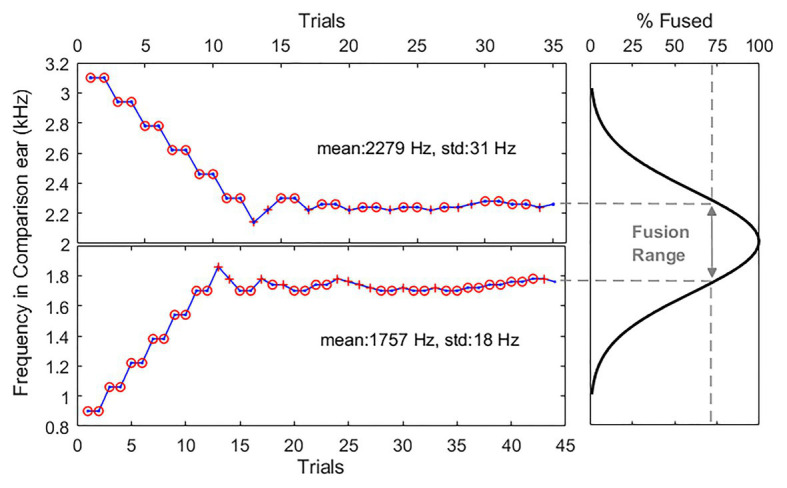
Example adaptive tracking results for one representative subject in the quiet condition. Left panels show two separate interleaved tracks in one testing block. Upper panel and lower panel show the adaptive tracking results when the comparison tone frequencies were presented above and below than the 2-kHz reference, respectively. Circle and cross symbols indicate different (not fused) and same (fused) responses, respectively. Right panel shows an estimated fusion function (thick solid line) and a fusion range (line with double arrows) calculated as the frequency offset between the two thresholds with 70.7% confident.

Figure 1 shows example two-down-one-up adaptive tracking results (left two panels) and an estimated fusion function (right panel) for one representative subject, N2, at the 2-kHz reference frequency. Initial frequency difference was set at 2000 ± 1109Hz between two ears and decreased in frequency by 160Hz after two consecutive “different” responses (“o” symbols) and increased after one “same” response (“+” symbols) in the following trial. The step sizes were reduced by a factor 2, and the minimum step size was fixed at 20Hz. Each run continued until there were a total of 10 reversals, with the first four reversals discarded. The perceptual fusion threshold was estimated as the geometric mean of the last six reversals. The fusion range was calculated as the frequency offset between the two thresholds (dashed lines), defining the range of frequency over which the subjects remained more than 70.7% confident that the dichotic stimulus was perceptually fused. Note that if subjects heard a sound only in one ear (lateralization), they were instructed to indicate that they heard one sound, as lateralization provides additional support for a fused percept. Only two subjects reported such lateralized fused perception in some paired reference-comparison tone frequencies. This lateralized perception might be due to inaccuracies in the loudness balancing procedure.

Fusion range measurements were collected in different background listening conditions: (1) quiet and (2) three different levels of steady pink noise. The 2000-ms steady pink noises were generated with cutoff frequencies of 200Hz and 8,000Hz. The overall noise levels were set in signal-to-noise ratio (SNR) of +15dB SNR, +5dB SNR, and −5dB SNR, where 1/3-octave filter outputs in the noise were equalized with the comfortable tone levels estimated from the loudness balancing procedure. The noises were gated on 250ms prior to onset of the tone stimuli, and remained on for 1,750ms, and presented dichotically. This ensured that the tone signals were always maintained in the middle of the steady background pink noise. It should be noted that the three SNR conditions were determined by preliminary fusion range measurements with the first three subjects (N1, N2, and N3). For all subjects, the noise levels were slightly different between two ears because the loudness-balanced tone levels were asymmetric across ears. In addition, pink noise was used in this study because the spectrum of pink noise closely matches the broad range of sounds in everyday listening environments, and it is widely used for auditory therapy such as tinnitus and hyperacusis treatments. The results for all experiments were averaged with two separate runs for each condition. All statistical analyses were conducted on the octave-scale data in SPSS (version 25, IBM).

## Results

### Binaural Pitch Fusion Ranges Are Symmetric Across the Ears in the Quiet Condition

**Figure 2:**
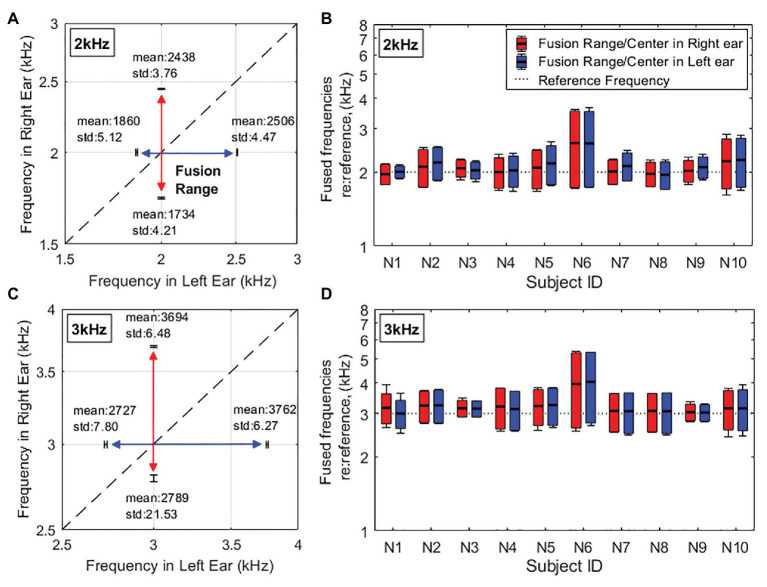
Fusion range results in the quiet [∞ dB signal-to-noise ratio (SNR)] condition. **(A,C)** Example two-dimensional representations of fusion range results for one representative subject (N2) at the reference frequency of 2kHz **(A)** and 3kHz **(C)**. The diagonal dashed line indicates the same frequency between two ears. **(B,D)** Individual fusion range results at the reference frequency of 2kHz **(B)** and 3kHz **(D)** indicated by horizontal dotted lines. The red lines/bars show fusion ranges in the right ear when the left ear is presented as the reference ear. The blue lines/bars show fusion ranges in the left ear when the right ear is presented as the reference ear. Error bars of endpoints represent across-trial SDs. The horizontal solid lines inside the boxes show the fusion centers.

Figures 2A,C illustrate the two-dimensional representation of fusion ranges for one representative subject, N2, at 2-kHz and 3-kHz reference frequencies, respectively. The colored double-arrow lines correspond to what range of frequencies in the contralateral ear were fused with a constant tone (2 or 3kHz) in the reference ear. In the given example in the figure, it is important to note that the fusion ranges mapped out on the two-dimensional figure are not aligned exactly around the dashed line, which represents the same frequency between two ears. Instead, the fusion range is shifted up toward the high frequencies in each ear. This means that more of the higher frequencies above the reference frequency are fused than the frequencies below the reference frequency. This example is representative as all collected fusion data show the same shift into the high frequencies across all subjects.

Figures 2B,D are a bar graph representation of individual pitch fusion results in the quiet condition (meaning no background noise was presented) at 2- and 3-kHz reference frequencies, respectively. Each vertical bar represents the upper and lower boundaries of the fusion range and the fusion center. Note that the fusion center results were used as a measure of the overall frequency offset of the fusion range relative to the reference frequency and were calculated as the weighted average of the frequencies within the fusion range. Although there is variability in fusion ranges between subjects, the fusion ranges across the ear are relatively symmetric meaning that for an individual, the fusion range measured in their left ear at one reference frequency (2 or 3kHz) will be the approximately same as that in the right ear. Averaged fusion ranges in the quiet condition are relatively symmetric across the two ears (2-kHz reference frequency: 797 ± 79 and 819 ± 78Hz; 3-kHz reference frequency: 1220 ± 110Hz and 1216 ± 117Hz in left and right ears, respectively). In addition, averaged fusion center results shows that fusion ranges were shifted toward to the higher frequencies than the references (fusion centers at 2-kHz reference: 2108 ± 187Hz and 2146 ± 179Hz; at 3-kHz reference: 3228 ± 253Hz and 3212 ± 287Hz in left and right ears, respectively).

### Steady Background Pink Noise Sharpens Binaural Pitch Fusion

**Figure 3:**
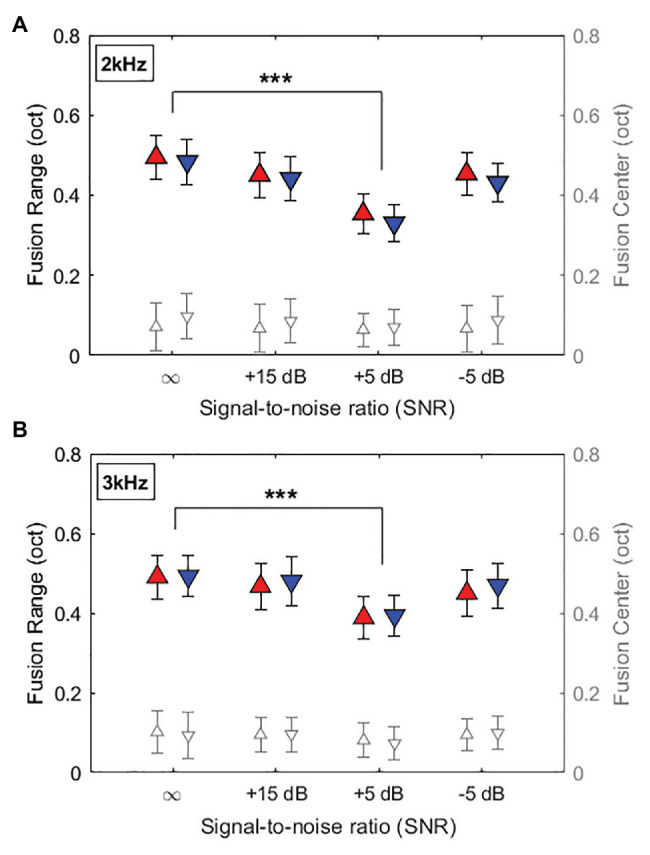
Averaged fusion range and fusion center results at the reference frequency of 2kHz **(A)** and 3kHz **(B)** as a function of SNR (∞, +15, +5, and −5dB SNR). ∞ indicates no background noise condition. Filled symbols and open symbols indicate fusion ranges in the left y-axis labels and fusion centers in the right y-axis labels, respectively. Red and blue colors represent fusion ranges in right and left ears, respectively. Error bars represent standard deviations of the mean. Asterisk symbols at the top indicate significant differences in fusion ranges (^***^p < 0.001).

Figure 3 shows the averaged fusion ranges on an octave scale at 2-kHz and 3-kHz reference frequencies as a function of SNR (∞, +15, +5, and −5dB SNR). At the 2-kHz reference frequency in Figure 3A, mean fusion ranges (filled symbols) in each ear continued to decrease with decreased SNRs (increased noise levels), minimized at +5dB SNR, and increased back at −5dB SNR (fusion range: 0.495, 0.451, 0.354, and 0.454 octaves in the right ear; 0.484, 0.441, 0.330, and 0.432 octaves in the left ear for ∞, +15, +5, and −5dB SNRs, respectively). A similar trend was also observed in the 3-kHz reference frequency condition (fusion range: 0.491, 0.467, 0.389, and 0.450 octaves in the right ear; 0.492, 0.480, 0.393, and 0.470 in the left ear for ∞, +15, +5, and −5dB SNRs, respectively).

A three-way RM-ANOVA was performed with fusion ranges as the dependent variable, and listening condition (quiet and three steady background noise conditions), reference ear (left and right), and reference frequency (2 and 3kHz) as within-subject factors. The results showed significant main effect of listening condition on fusion ranges (*F*_3,144_ = 10.56, *p* < 0.001, *η*^2^ = 0.19), but no main effects of the reference ear and reference frequency and no significant interaction between these factors. *Post hoc* pairwise comparisons using Bonferroni correction performed to better understand the main effect of listening condition. Relative to the quiet condition, adding the +5-dB SNR steady background noise in which the noise level was 5dB lower than the signals significantly decreased binaural fusion ranges for both 2- and 3-kHz references in each ear (*p* < 0.001 for all cases).

Figure 3 also shows the overall offset of the fusion range relative to the reference frequency in each listening condition. In both 2 and 3-kHz reference frequencies, mean fusion centers were varied from 0.06 to 0.1 octaves higher than the reference frequencies, regardless of the reference ears and noise levels. Results from a RM-ANOVA with fusion center as the dependent variable showed no significant main effects of listening condition, reference ear, and reference frequency on the fusion center shifts (*p* > 0.1 for all cases). However, separate one-sample *t*-tests showed significant offsets of fusion centers from the reference frequency (*p* < 0.01 for all cases).

## Discussion

The purpose of this study was to investigate how binaural pitch fusion can be influenced by soft but audible external noise. The averaged data show that the addition of steady background pink noise to the task, at +5dB SNR, suppresses pitch fusion ranges which indicate sharpening binaural pitch fusion, but does not affect fusion-center shift. It is important to note that variability in the effects of noise was also observed even with small sample sizes (*N* = 10). As with hearing levels, the specific levels of noise required to elicit the narrowing of fusion ranges varied. Some subjects (3 out of 10) showed a greater benefit across each trial, while others may have seen improvement with either a higher or lower SNR. Some subjects (4 out of 10) also showed broadened fusion ranges at the lower SNR (−5dB SNR), in which the background noise might be too loud to discriminate pitch differences across ears (i.e., masking effect). These findings suggest that the window where they received benefit from external noise before it causes masking effects will be non-monotonic across subjects. However, despite this individual variability, the trend in averaged data is clear in showing that there is a change to fusion ranges from quiet where individuals will receive a benefit before the masking effect begins. Further studies with the finer resolution of SNRs may yield realistic estimates of the boundary between benefit and masking effects and provide a better understanding of how steady noises affects binaural pitch fusion.

Possible mechanisms of stochastic resonance have been explored in various studies for auditory perception mostly in CI users. A common explanation from previous studies is that CI users have lower than normal stochastic nerve activity and abnormal across-fiber synchrony due to the absence of hair cells that, in a healthy auditory system, generate noise spontaneously in quiet. Adding background noise in either subthreshold or suprathreshold levels can help improve transmission of information by reducing across-fiber synchrony thus mimicking the spontaneous activity observed in a healthy cochlea (Zeng et al., 2000; Chatterjee and Robert, 2001; Allingham et al., 2003; Paglialonga et al., 2010). This stochastic resonance effect was also theorized to be effective in the projection site of the auditory nerve at the level of the dorsal cochlear nucleus. Krauss et al. (2016) hypothesized that damage to the cochlea, resulting in a diminished number of inner hair cells converging onto dorsal cochlear nucleus neurons, would result in sub-threshold auditory nerve input that would not be sufficient to evoke a response. Therefore, the background noise that is needed for stochastic resonance to take place could be generated by spontaneous activity generated in the dorsal cochlear nucleus.

The findings in this study (that the addition of soft but audible noise to a normal auditory system still yields improvement) suggest an interesting idea that the auditory system may not be harnessing its own stochasticity at maximum efficiency, especially in binaural pitch perception performance. This mirrors a suggestion from Zeng et al. (2000), who presented improvements in thresholds saying “Although the improvement in thresholds by noise is relatively small (1.4–1.7dB) in normal-hearing subjects, it suggests that the normal auditory system, while possibly already using the stochastic resonance in hearing, is not optimal” (Zeng et al., 2000). In line with the results of Zeng et al. (2000), could the improvement seen in the current study by NH individuals be more drastic when repeated with individuals with hearing loss?

Our current study about binaural pitch fusion is focused on how two dichotic information paths integrate with each other. This effect can be explained by being a peripheral process, where each side has their own benefit, or a central process where the brainstem or cortex could be affected by stochastic resonance. This distinction on the mechanism of stochasticity is still unknown and not clarified by the current study. The current study only focuses on effects of soft but audible external noise on binaural pitch fusion in NH listeners. Further studies into whether the same effect takes place, and if it takes place at the same SNR, in HI listeners are the next avenue to explore. Given the broader binaural fusion present in HI individuals, these specific SNRs may not cause the same sharpening effect. Instead, a different SNR could be more optimal based on the specific subject’s hearing thresholds. It should be noted that this paper reports preliminary results with only 10 NH subjects, therefore it is speculated that more complicated stochastic resonance effects may be observed in the hearing-impaired listeners. Evaluations of more NH subjects are required to understand how this stochastic resonance effect can practically be used to help improve binaural auditory perception. In addition, studying this further may lead to another path for intervention to help with the rehabilitative care of hearing-impaired listeners. As proposed by Oh et al. (2019), abnormally broad binaural fusion which is often observed in hearing-impaired listeners could interfere with the segregation of auditory objects based on pitch differences, such as multiple voices in a multi-talker listening environment. Increased understanding of abnormal binaural fusion in hearing-impaired listeners can provide information for future design and training with device-based rehabilitative strategies to enhance the benefits of binaural processing for speech perception in noise.

## Conclusion

To our knowledge, this is the first study to systematically investigate how binaural pitch fusion (pitch discrimination across the ears) can be influenced by the presence of external noise. The preliminary findings in normal hearing listeners show that the addition of steady background pink noise at 5-dB SNR can reduce binaural pitch fusion ranges, which illustrates an effect called stochastic resonance. Although the degree of individual variability in the effects of noise was observed, the trend in averaged data is clear in showing that specific noise levels can sharpen binaural pitch fusion. This finding suggests that the stochastic resonance effect could be used to further study potential rehabilitation approaches that reduce broad binaural fusion ranges, like those that are often observed in hearing impaired listeners.

## Data Availability Statement

The original contributions presented in the study are included in the article/supplementary material, further inquiries can be directed to the corresponding author.

## Ethics Statement

The studies involving human participants were reviewed and approved by Institutional Review Board of University of Florida. The patients/participants provided their written informed consent to participate in this study.

## Author Contributions

YO designed the experiments. SL performed the experiments. YO and SL analyzed the data, wrote the article, and discussed the results at all states. All authors contributed to the article and approved the submitted version.

### Conflict of Interest

The authors declare that the research was conducted in the absence of any commercial or financial relationships that could be construed as a potential conflict of interest.
